# Patterns and factors associated with electrolyte abnormalities among patients with heart failure in Uganda

**DOI:** 10.1186/s12872-024-04276-1

**Published:** 2024-11-05

**Authors:** Awil Abdulkadir Abdi, Nyende Louis, Abshir M. Hirsi, Ibrahim Ahmed Nur, Muktar Hassan Mohamud, Wardat Rashid Ali, Naqeeb Kara Imtiaz

**Affiliations:** Department of Internal Medicine, Faculty of Clinical Medicine and Dentistry, Kampala International University, Ishaka, Bushenyi, Uganda

**Keywords:** Heart failure, Electrolyte abnormalities, Drug adherence, Diuretic use, Hypertension

## Abstract

**Background:**

Electrolyte abnormalities (EAs) worsen the clinical course of patients with heart failure (HF). The patterns of EAs vary among patients with HF. This study investigated patterns and factors associated with EAs among patients with HF admitted to Hoima Regional Referral Hospital (HRRH) in western Uganda.

**Methods:**

This hospital-based cross-sectional study used quantitative data of 384 HF patients admitted at HRRH between 21st February and 15th May 2023. Data on sociodemographic, lifestyle, and medical characteristics were collected and presented as descriptive statistics. EAs were considered electrolyte values below or above the reference normal ranges. Bivariate and multiple logistic regression analyses were conducted to establish associations. An association with a *p* < 0.05 is considered statistically significant.

**Results:**

Of 384 HF patients, 342 (89.1%) had EAs. Hypocalcemia was the most common EA, 165 (43.0%). Among the patients, 69 (18.0%) were on diuretics, 185 (48.2%) were on angiotensin-converting enzyme inhibitors (ACEIs)/angiotensin receptor blockers (ARBs), and 105 (27.3%) were on calcium channel blockers (CCBs). Additionally, 264 (68.8%) had a history of hypertension, and 20 (5.2%) demonstrated good drug adherence. Patients with good drug adherence had lower odds of EAs (Adjusted Prevalence Odds Ratio [Adjusted POR] = 0.2, 95% CI: 0.1–0.7, *p* = 0.009). Those on diuretics had higher odds of EAs compared to those on ACEIs/ARBs and CCBs, with an Adjusted POR of 5.7 (95% CI: 1.3–15.0, *p* = 0.019). A history of hypertension also increased the odds of EAs (Adjusted POR = 4.0, 95% CI: 1.9–8.4, *p* < 0.001).

**Conclusions:**

The prevalence of EAs in patients with HF at HRRH was high, with hypocalcemia being the most common. Patients with good drug adherence had lower odds of EAs. On the other hand, diuretic use and a history of hypertension were associated with increased odds of EAs.

## Introduction

Heart failure (HF) refers to the heart’s inability to work as a pump, failing to maintain an adequate blood flow to the tissues under various physiological conditions [1]. HF affects more than 64.3 million people worldwide [2]. This number is expected to rise due to an increase in the average life expectancy of the general population [3]. In sub-Saharan Africa, HF accounts for approximately 9.4–42.5% of all medical admissions and approximately 25.6–30.0% of admissions to cardiac units [4].

Electrolyte abnormalities (EAs) are common among patients with HF due to the complications of HF itself and various medications used for treatment. These abnormalities are often underestimated or undiagnosed in many healthcare settings [5]. For instance, hypokalaemia and hypomagnesemia contribute to 50% of all sudden fatalities from HF and ventricular arrhythmias [6], and increased potassium levels were associated with a high risk of all-cause death among hospitalized patients with HF [7]. Hypochloremia in HF is associated with a poor diuretic response, an increased need for inotropes, and a high risk of mortality during hospitalization [8]. The abnormalities of magnesium, phosphate, and calcium were linked to HF complications including atrial fibrillation and myocardial ischemia [9].

Globally, the prevalence of EAs among patients with HF varies. The most common disturbed electrolytes include sodium, chloride, and potassium [10]. In Denmark, 32.6% of HF patients had hypocalcemia, and 4.3% had hypercalcemia [11]. In Austria, elevated phosphate levels were observed in 5.8–6.0% of HF patients [12]. While in Indonesia, 22% of HF patients had hyponatremia, 10% had hypokalemia, and 7% had hyperkalemia [13]. Hyponatremia is a commonly reported abnormality among patients with HF in most studies [14].

In Africa, studies have shown varying rates of EAs in HF patients. In Cameroon, 26.2% of hypertension patients were hypokalemic, with 3.9% being HF patients [15]. In Ethiopia, 27.6% of HF patients had hypokalemia and 6.0% had hyperkalemia [16], while another study found that 28.1% of HF patients had at least one EA [17]. In Uganda, 24.2% of elderly HF patients had hyponatremia [5].

The purpose of this study is to document the patterns and factors associated with EAs in patients with HF admitted to HRRH and in Uganda, where currently no data on EAs exist. This study aims to fill that gap and provide valuable insights to improve patient management.

## Methods and materials

### Study design

This cross-sectional study was conducted in the Internal Medicine Ward and Hypertension Clinic of HRRH between 21st February and 15th May 2023. Participants were enrolled using a consecutive sampling technique.

## Study site

The study was conducted at HRRH, where blood samples were analyzed in the hospital laboratory for electrolytes. This was a single-center study.

## Study population

The study included patients with HF, both previously diagnosed and newly diagnosed, who were recruited from the Internal Medicine Ward and Hypertension Clinic at HRRH.

## Inclusion criteria

**Table:** 

Major Criteria	Minor Criteria
Paroxysmal Nocturnal Dyspnea or Orthopnea	Tachycardia > 120 bpm
Raised Jugular Venous Pressure	Orthopnea
S3 Gallop	Exertional Dyspnea
Cardiomegaly	Nocturnal cough
Hepatojugular Reflex	Ankle edema
Pulmonary Edema	Hepatomegaly
Rales	Pleural effusion

Participants were eligible for inclusion if they were known or newly diagnosed with HF using Framingham criteria, were ≥ 18 years of age, and consented to participate in the research. Framingham’s Diagnostic Criteria for HF has a sensitivity of 92% and a specificity of 89%^18^. Two majors or one major and two minors were diagnostic.

## Exclusion criteria

The study excluded patients who were taking medications known to cause EAs, like amphotericin B or carbamazepine.

### Sample size

The sample size for the prevalence of EAS among patients with HF was determined using Daniel formula as illustrated below [19].

$$\:n=\:\frac{{z}^{2}\:p\left(1-p\right)}{{d}^{2}}$$

Where, n = Minimum sample size.

Z = The table value for standard normal deviation corresponding to 95% significance level (= 1.96), P = Prevalence of characteristic being estimated, d = Margin error, set at 0.05.

The sample size for this study was determined by calculating the predicted prevalence of 50%, as no previous study had been conducted in the local regions. The value utilized for P in the calculation was 0.5.

$$\:n=\:\frac{{\left(1.96\right)}^{2}\:0.5\left(1-0.5\right)}{{\left(0.05\right)}^{2}}\:n=\:\frac{3.8416\:x\:0.5\:x\:0.5}{0.0025}\:n=\:384$$

## Data collection procedure and recruitment

All necessary precautions and mitigation measures for Ebola and COVID-19 were followed throughout the research. My team and I screened patients in the Internal Medicine Ward and Hypertension Clinic at HRRH. Data was collected during patient admission in the internal medicine ward from 9:00 am to 5:00 pm on working days and during hypertension clinic visits every Thursday from 9:00 am to 5:00 pm. Participants who met the inclusion criteria were informed about the study’s objectives, and written consent was obtained before data collection. Data collection involved administering structured questionnaires for demographic and clinical information and obtaining blood samples for electrolyte assessment.

Clinical examinations, conducted by the principal investigator and a qualified medical doctor, included measurements of body mass index (BMI), calculated by dividing weight (kg) by squared height (m²). Around 5 mL of blood was collected from each participant for laboratory evaluation by a qualified laboratory technician. An intern nurse promptly transported the samples to the hospital laboratory within 10 min of collection. The hospital laboratory technicians analyzed them at the hospital’s chemistry laboratory using a Cobas C311 analyzer. Daily quality control checks were performed using a system check cartridge designed to ensure the absorbance readings’ accuracy and standard sample analyses.

## Study variables

### Independent variables

Non-Modifiable Factors: Age, sex, education, marital status, religion.

Modifiable Factors: Smoking, alcohol use, physical activity, and salt intake.

Medical Factors: body mass index (BMI), Medications of HF, duration of HF, drug adherence, diabetes mellitus (DM), hypertension (HTN), HIV status, chronic kidney disease, New York Heart Association (NYHA) classification.

Medication adherence was evaluated using the Morisky Medication Adherence Scale (MMAS-8) which has 8 questions, and focuses on a patient’s medication experience over the prior two weeks. Seven of the eight questions have a simple yes/no format. The last question uses a 5-point Likert scale. All items were coded as “no” = 0 and “yes” = 1, except for item 8. In item 8, if a patient selects “0,” the score is recorded as “1,” and the remaining 4 scores are recorded as “0. A score < 6 indicates poor adherence(low), a score between 6 and 7 indicates fair adherence (medium) and a score of 8 indicates good adherence (high) [20, 21].

1. Do you sometimes forget to take your medications? [yes (0), No (1)]
2. People sometimes miss taking their medications for reasons other than forgetting. Thinking over the past two weeks, were there any days when you did not take your medicines? [yes (0), No (1)]
3. Have you ever cut back or stopped taking your medication without telling your doctor, because you felt worse when you took it? [yes (0), No (1)]
4. When you travel or leave home, do you sometimes forget to bring along your medications? [yes (0), No (1)]
5. Did you take your medications yesterday? [yes (0), No (1)]
6. When you feel like your condition is under control, do you sometimes stop taking your medicines? [yes (0), No (1)]
7. Taking medication every day is a real inconvenience for some people. Do you ever feel hassled about sticking to your treatment plan [yes (0), No (1)]
8. How often do you have difficulty remembering to take all your medications? Never/rarely (1), Once in a while (0), Sometimes (0) Usually (0), All the time (0).

### Dependent variable

Electrolyte abnormalities were defined as values below or above the normal range for the respective electrolytes. The normal ranges for serum electrolytes were: sodium (135–145 mmol/L), potassium (3.5–5.5 mmol/L), chloride (98–107 mmol/L), calcium (2.15–2.50 mmol/L), phosphate (0.18–1.45 mmol/L), and magnesium (0.66–1.07 mmol/L). Abnormal values were defined as follows: sodium < 135 mmol/L (hyponatremia) or > 145 mmol/L (hypernatremia), potassium < 3.5 mmol/L (hypokalemia) or > 5.5 mmol/L (hyperkalemia), chloride < 98 mmol/L (hypochloremia) or > 107 mmol/L (hyperchloremia), calcium < 2.15 mmol/L (hypocalcemia) or > 2.5 mmol/L (hypercalcemia), phosphate < 0.18 mmol/L (hypophosphatemia) or > 1.45 mmol/L (hyperphosphatemia), and magnesium < 0.66 mmol/L (hypomagnesemia) or > 1.07 mmol/L (hypermagnesemia).

EAs were categorized into different patterns based on the results. A single hypo or hyper refers to an abnormality in one electrolyte, either low or high. A mixed pattern was defined as the presence of more than one electrolyte abnormality, which could involve different electrolytes or a combination of both low and high levels across several electrolytes.

### Quality control

The data collection tool was pretested for validity and reliability, and adjustments were made before data collection. Research assistants were trained for the study objectives. The principal investigator double-checked the data daily for accuracy. Quality control measures included regular calibration of laboratory equipment, and random samples were sent to an external laboratory (Lancet Laboratory of Hoima district, Uganda; an officer took those samples to the Lancet Laboratory with appropriate transportation) by comparing them to the hospital chemical laboratory for validation.

### Data management and analysis

The data was coded, cleaned, and entered into Microsoft Excel Office 2021 and then exported to STATA© 17 for analysis. Descriptive statistics were used to summarize the prevalence and patterns of EAs. Bivariate and multivariable logistic regression analyses were conducted and the variables with *p-value* < 0.2 or crude Prevalence Odds ratio (CPOR) ≥ 2 or ≤ 0.5 at bivariate analysis were entered into a multivariable model. A factor was considered significant at *p* < 0.05.

### Ethical considerations

All methods were carried out following relevant guidelines and regulations. Ethical approval was granted by the Institutional Research and Ethical Committee (IREC) of Kampala International University (REC NO: KIU-2022-204). All participants provided written informed consent. The study adhered to COVID-19 and Ebola SOPs, ensuring the safety of participants and researchers. Privacy and confidentiality were maintained using unique identifiers, and data storage was secure.

## Results

Three hundred and eighty-four patients with HF at HRRH were analyzed during the study period between 21st February and 15th May 2023.

Of the 384 HF patients, 69.8% (*n* = 268) were female, with a mean age of 61 years (SD = 10.2). Most were self-employed (78.6%, *n* = 302), had formal education (73.7%, *n* = 283), and engaged in ≥ 30 min of physical activity daily (68.0%, *n* = 261). Hypertension was present in 68.8% (*n* = 264). The majority reported poor drug adherence (88.5%, *n* = 340), a HF duration of < 6 months (88.4%, *n* = 327), and treatment duration of < 6 months (88.5%, *n* = 328) (Table 1).

**Table 1:** Sociodemographic, lifestyle, and medical characteristics of heart failure patients who underwent HRRH (*n* = 384)

Sociodemographic and lifestyle characteristics		*N* (%)
Gender	Male	116 (30.2)
	Female	268 (69.8)
Age (Years) M = 61.0, SD = 10.2	< 60	162 (42.2)
	≥ 60	222 (57.8)
Formal education	No	101 (26.3)
	Yes	283 (73.7)
Religion	Christians	315 (82.0)
	Muslims	69 (18.0)
Resident	Hoima city	330 (85.9)
	Others	54 (14.1)
Occupation	Employee	54 (14.1)
	Self -employed	302 (78.6)
	Unemployed	63 (16.4)
Marital status	Single	30 (7.8)
	Married	169 (44.0)
	Separated/Divorced	107 (27.9)
	Widowed	78 (20.3)
Physical activity	< 30 min per day	123 (32.0)
	≥ 30 min per day	261 (68.0)
Current Smoking	No	361(94.0)
	Yes	23(6.0)
Current Alcohol	No	256(66.7)
	Yes	128(33.3)
BMI	Normal	206 (53.6)
	Underweight	26 (6.8)
	Overweight/obese	152 (39.6)
Diabetes Mellitus	No	277 (72.1)
	Yes	107 (27.9)
HIV	No	347(90.4)
	Yes	37 (9.6)
Hypertension	No	120 (31.3)
	Yes	264 (68.8)
Chronic Kidney Disease	No	363 (94.5)
	Yes	21 (5.5)
Stroke	No	372 (96.9)
	Yes	12 (3.1)
Severity of HF (NYHA)	I-II	322(83.9)
	III-IV	62(16.1)
HF duration (months)	< 6	327 (85.2)
	≥ 6	57 (14.8)
Duration of treatment (months)	< 6	328 (85.4)
	≥ 6	56 (14.6)
Drug adherence	Poor	340 (88.5)
	Fair	24 (6.3)
	Good	20 (5.2)
Regimens received	ACE/ARBs	185 (48.2)
	CCBs	105 (27.3)
	Diuretics	69 (18.0)
	Others	25 (6.5)

BMI: body mass index, HF: heart failure ACEi: angiotensin convertase enzyme inhibitors, ARBs: angiotensin receptor blockers

### Patterns of electrolyte abnormalities among patients with heart failure attending HRRH

Of the 384 patients with HF enrolled, the majority presented with mixed EAs (53.9%). Hypocalcemia was the most common abnormality, occurring in 43.0% of the patients (Table 2).

**Table 2:** Patterns of electrolyte abnormalities among patients with heart failure who underwent HRRH (*n* = 384)

		Total	Age (years)	
Electrolyte		**n (%)**	< 60	≥ 60
Potassium	Normokalaemia	327 (85.2)	127(38.8)	200(61.2)
	Hypokalemia	34 (8.9)	18 (52.9)	16 (47.1)
	Hyperkalemia	23 (6.0)	17 (73.9)	6 (26.1)
Sodium	Normonatremia	238 (62.0)	97(40.8)	141(59.2)
	Hyponatremia	122 (31.8)	53 (43.4)	69 (56.6)
	Hypernatremia	24 (6.3)	12 (50.0)	12 (50.0)
Magnesium	Normomagnesemia	217 (56.5)	89(41.0)	128(59.0)
	Hypomagnesaemia	155 (40.4)	67 (43.2)	88 (56.8)
	Hypermagnesemia	12 (3.1)	6 (50.0)	6 (50.0)
Calcium	Normocalcaemia	184 (47.9)	82(44.6)	102(55.4)
	Hypocalcemia	165 (43.0)	60 (36.4)	105 (63.6)
	Hypercalcemia	35 (9.1)	20 (57.1)	15 (42.9)
Phosphate	Normophosphatemia	327 (85.2)	143(43.7)	184(56.3)
	Hypophosphatemia	43 (11.2)	14 (32.6)	29 (67.4)
	Hyperphosphatemia	14 (3.6)	5 (35.7)	9 (64.3)
Chloride	Normochloremia	310 (80.7)	132(42.6)	178(57.4)
	Hypochloremia	18 (4.7)	6 (33.3)	12 (66.7)
	Hyperchloremia	56 (14.6)	24 (42.9)	36 (64.3)
Overall, EA	None	42(10.9)	16 (38.1)	26 (61.9)
	*Single - Hypo	107 (27.9)	43 (40.2)	64 (59.8)
	*Single - Hyper	28 (7.3)	12 (42.9)	16 (57.1)
	Mixed	207 (53.9)	91 (43.9)	116 (56.1)

* Single ‘hypo’ or ‘single hyper’ refers to a single occurrence of either low or high levels of any electrolyte.

*Mixed: the occurrence of more than one electrolyte abnormality, either involving different electrolytes or a combination of hypo and hyper states in multiple electrolytes.

The overall prevalence of EAs in patients with HF was 342 (89.1%) (95% CI: 85.5–91.8).

Table 3 shows the bivariate analysis of factors associated with EAs among HF patients. Variables with a p-value < 0.2 or a Crude Prevalence Odds Ratio (CPOR) ≥ 2 or ≤ 0.5 were entered into the multivariable model for further analysis.

**Table 3:** Bivariate results of the factors associated with EA among patients with HF attending HRRH (*n* = 384)

Factors		EAs (*N* = 384)		Crude P0R (95%CI)	P
		No (*n* = 42)	Yes (*n* = 342)		
Gender	Male	8 (19.0)	108 (31.6)	1	
	Female	34 (81.0)	234 (68.4)	0.5(0.2–1.1)	0.100
Age (Years)	< 60	15 (35.7)	147 (43.0)	1	
	≥ 60	27 (64.3)	195 (57.0)	0.7(0.4–1.4)	0.369
Formal education	No	13 (31.0)	88 (25.7)	1	
	Yes	29 (10.3)	254 (89.7)	1.2(0.6–2.6)	0.720
Religion	Christians	37 (11.8)	278 (88.2)	1	
	Muslims	5 (7.3)	64 (92.7)	1.7(0.5–2.4)	0.283
Residence	Hoima city	34 (81.0)	296 (86.5)	1	
	Others	8 (19.0)	46 (13.5)	0.7(0.3- 1.5)	0.328
Occupation	Employe	6 (9.5)	57 (90.5)	1	
	Self-employed	31 (10.3)	271 (89.7)	0.9(0. 4-2.4)	0.859
	Unemployed	5(26.3)	14(73.7)	0.2(0.1–1.1)	0.070
Marital status	Single	1 (2.4)	29 (8.5)	1	
	Married	18 (42.9)	151 (44.2)	0.3(0.0-2.3)	0.236
	Separated	14 (33.3)	93 (27.2)	0.2(0.0-1.8)	0.163
	Widowed	9 (21.4)	69 (20.2)	0.3(0.0-2.2)	0.217
Salt intake	No	15 (35.7)	155 (45.3)	1	
	Yes	27 (64.3)	187 (54.7)	0.7(0.3–1.3)	0.239
BMI	Normal	24 (57.1)	182 (53.2)	1	
	Underweight	2 (4.8)	24 (7.0)	1.6(0.4–7.1)	0.550
	Overweight/Obese	16 (38.1)	136 (39.8)		
Physical activity	< 30 min per day	16 (38.1)	107 (31.3)	1	
	≥ 30 min per day	26 (61.9)	235 (68.7)	1.4(0.7–2.6)	0.373
Diabetes Mellitus	No	33 (78.6)	244 (71.3)	1	
	Yes	9 (21.4)	98 (28.7)	1.3(0.7–3.2)	0.327
HIV	Negative	38 (90.5)	309 (90.4)	1	
	Positive	4 (9.5)	33 (9.6)	1.2(0.4–3.7)	0.715
Hypertension	No	26(21.7)	94(78.3)	1	
	Yes	16(6.1)	248(93.9)	4.3(2.2–8.3)	< 0.001***
Chronic Kidney disease	No	41 (97.6)	322 (94.2)	1	
	Yes	1 (2.4)	20 (5.8)	2.5(0.3–19.5)	0.368
Stroke	No	41 (97.6)	331 (96.8)	1	
	Yes	1 (2.4)	11 (3.2)	1.4(0.2–10.8)	0.770
Severity of HF (NYHA)	I-II	38 (90.5)	284 (83.0)	1	
	III-IV	4 (9.5)	58 (17.0)	1.94(0.7–5.6)	0.224
HF duration (months)	< 6	37 (88.1)	290 (84.8)	1	
	≥ 6	5 (11.9)	52 (15.2)	1.3(0.5–3.5)	0.571
Treatment duration (months)	< 6	36 (85.7)	292 (85.4)	1	
	≥ 6	6 (14.3)	50 (14.6)	1.0(0.4–2.6)	0.954
Drug adherence	Poor	33 (78.6)	307 (89.8)	1	
	Fair	3 (7.1)	21 (6.1)	0.8(0.2–2.7)	0.659
	Good	6 (14.3)	14 (4.1)	0.3(0.1–0.7)	0.008*
Drug regimen	ACE/ARBs	29 (69.0)	156 (45.6)	1	
	CCBs	9 (21.4)	96 (28.1)	2.0(0.9–4.4	0.089
	Diuretics	2 (4.8)	67 (19.6)	6.2(1.4–16.8)	0.014*
	Others	2 (4.8)	23 (6.7)	2.1(0.5–9.6)	0.320
Vomiting	No	38 (90.5)	320 (93.6)	1	
	Yes	4 (9.5)	22 (6.4)	0.7(0.2-2.0)	0.455
Current Smoking	No	42(100.0)	319(93.3)	1	
	Yes	0(0.0)	23(6.7)	N/A	
Current Alcohol	No	30(71.4)	226(66.1)	1	
	Yes	12(28.6)	116(33.9)	1.3(0.6–2.6)	0.489

*p < 0.05. **p < 0.01. ***p < 0.001.

Crude POR: Crude Prevalence Odds Ratio, BMI: body mass index, HF: heart failure, ACEi: angiotensin convertase enzyme inhibitors, ARBs: angiotensin receptor blockers, NYHA: New York Heart Association.

The results from the multivariable model in Table 4 indicate that patients with HF who had good drug adherence had lower odds of EAs (Adjusted POR = 0.2, 95% CI: 0.1–0.7, *p* = 0.009). Patients receiving diuretics had higher odds of EAs than those on ACEi/ARBs and calcium channel blockers (Adjusted POR = 5.7, 95% CI: 1.3–15.0, *p* = 0.019). Additionally, a history of hypertension was associated with greater odds of EAs (Adjusted POR = 4.0, 95% CI: 1.9–8.4, *p* < 0.001).

**Table 4:** Multivariable analysis of the factors associated with electrolyte abnormalities among patients with heart failure who underwent HRRH (*n* = 384)

Factors		Adjusted POR (95%CI)	P
Gender	Male	1	
	Female	0.7(0.3–1.7)	0.484
Occupation	Employe	1	
	Self-employed	1.1(0.4-3.0)	0.872
	Unemployed	0.4(0.1–2.1)	0.260
Marital status	Single		
	Married	0.4(0.1–3.2)	0.392
	Separated	0.3(0.0-2.5)	0.195
	Widowed	0.4(0.0-3.7)	0.235
Hypertension	No	1	
	Yes	4.0(1.9–8.4)	< 0.001***
Chronic kidney disease	No	1	
	Yes	1.4(0.2–11.4)	0.765
Drug adherence	Poor	1	
	Fair	0.6(0.2–4.8)	0.486
	Good	0.2(0.1–0.7)	0.009**
Drug regimen	ACE/ARBs	1	
	CCBs	2.2(0.9–5.1)	0.076
	Diuretics	5.7(1.3–15.0)	0.019*
	Others	5.8(0.6–12.0)	0.213

*p < 0.05. **p < 0.01. ***p < 0.001. Adjusted POR: Adjusted Prevalence Odds Ratio, BMI: body mass index, HF: heart failure, ACEi: angiotensin convertase enzyme inhibitors, ARBs: angiotensin receptor blockers, NYHA: New York Heart Association

## Discussion

This cross-sectional study conducted at HRRH among 384 patients with HF revealed an 89.1% prevalence of EAs, with hypocalcemia and hyponatremia being the most common. In comparison, studies from Pakistan and Ethiopia reported EAs in 53.1% and 34.5% of patients with HF, respectively, by investigating only sodium and potassium [22, 16].

In our study, 43% of the patients had hypocalcemia, which was higher compared to 32.6% in a Danish study of patients with chronic HF [11]. Hypercalcemia was present in 9.1% of our research, while the Danish study reported a lower prevalence of 4.3%^11^. Another study in Poland reported hypocalcemia and hypercalcemia in 22.9% and 8.7% of patients with HF, respectively [23].

The prevalence of hyponatremia in our study was 31.8%, compared to 24.2% in a study conducted by Nankabirwa et al. among elderly patients with HF [5]. A cross-sectional study of patients with HF conducted in Nepal revealed that most of the patients had hyponatremia, hypomagnesemia, hypocalcemia, and hyperphosphatemia, though specific prevalence for each electrolyte was not provided [24].

Our study investigated six electrolytes, yielding a higher prevalence due to the broader scope of electrolytes examined. This might be expected since most other studies separately assessed sodium, potassium, and chloride, or extended electrolytes, including calcium, magnesium, and phosphate.

Calcium homeostasis plays a pivotal role in myocardial contraction and relaxation, and several factors can contribute to calcium homeostasis abnormalities, including chronic kidney disease and diuretics [25]. Patients with low BMI had increased odds of having EAs at bivariate analysis in our study, although it was not statistically significant. A low dietary intake can result in hypocalcemia and vitamin D deficiency due to impaired gut absorption which are especially common in older adults or those with congestive HF, as intestinal congestion can impair nutrient absorption [23]. Loop diuretics increase calcium excretion, leading to hypocalcemia, while thiazide diuretics can cause hypercalcemia due to increased calcium reabsorption [26]. Therefore, serum calcium abnormalities can impair myocardial function, leading to electrical instability and negatively impacting HF prognosis [11].

Neurohormonal responses in HF, such as renin-angiotensin-aldosterone system (RAAS) and sympathetic nervous system activations, also contribute to EAs. These responses result in sodium and water retention (hyponatremia) and intracellularly shifting of potassium and magnesium causing hypokalemia and hypomagnesemia [6, 27.

These findings suggest that the varying patterns of EAs in patients with HF can be attributed to both the complications of HF itself and drug regimens.

In our study, patients with HF who had good drug adherence had lower odds of EAs, consistent with a multicenter study of patients with HF in South Korea, Taiwan, and China, reported that low drug adherence was associated with EAs [28]. A prospective study in Ethiopia also found that poor drug adherence was linked to EAs and poorer outcomes among patients with HF [16]. These findings indicate that drug adherence among patients with HF is crucial.

This study revealed that patients with a history of hypertension had increased odds of EAs. This aligns with findings from an Austrian study on serum phosphate in patients with HF [12]. Uncontrolled hypertension can damage arteries, leading to renal insufficiency and activating the RAAS system, resulting in sodium retention (hyponatremia) [29], and/or the effect of arteries on hypertension may cause sympathetic nervous system activation, which stimulates catecholamines that cause a shift of potassium and magnesium to intracellular levels with subsequent hypokalemia and hypomagnesemia [27].

The current study established that patients receiving diuretics had higher odds of EAs. Studies from Japan, Ethiopia, and Uganda consistently found that diuretic use in HF patients is associated with EAs, including dyskalemia and hyponatremia [30, 17, 5].

The most commonly used diuretics are loop and thiazide diuretics. The loop diuretics exert their effects within the ascending loop of Henle in the renal tubule by impeding the function of the sodium-potassium-chloride channel, promoting salt wasting and increasing calcium excretion, which leads to hyponatremia and hypocalcemia. On the other hand, thiazide diuretics act on the sodium-chloride cotransporter in the distal convoluted tubule, reducing sodium reabsorption [5, 26]. Both types of diuretics induce hypokalemia and hypomagnesemia by enhancing the flow rate of magnesium excretion in the loop of Henle [31, 32].

### Limitations and strengths of the study

This cross-sectional study was not designed to investigate the causality of EAs but rather to reveal their prevalence and factors associated with HF patients. One of the limitations of our study was the inability to measure corrected calcium levels due to financial constraints. Additionally, we could not perform echocardiography for HF diagnosis, which would have provided a better understanding of the cardiac function and its relationship with electrolyte abnormality. Despite these limitations, our findings highlight the novel and clinically relevant patterns of EAs, underscoring the importance of further research on multicenter and longitudinal studies and targeted clinical interventions.

## Conclusion

Monitoring standard and extended EAs in HF patients is crucial for improving outcomes. Ensuring good drug adherence is essential to reduce EAs.

## Data Availability

Data is available on request by this email: awil6263@gmail.com (primary investigator).

## Declarations

All the participants were requested to sign the consent form, as evidenced by the participants’ signature.
